# Development of a Novel Multiple Cross-Linking Spiral Amplification for Rapid and Sensitive Detection of HPV16 DNA

**DOI:** 10.4014/jmb.2012.12047

**Published:** 2021-01-22

**Authors:** Donghong Zhang, Dongliang Liu, Bing Liu, Xiulan Ma

**Affiliations:** Department of Otolaryngology Head and Neck Surgery, Shengjing Hospital of China Medical University, Shenyang 110004, P.R. China

**Keywords:** Human papillomavirus virus, multiple cross-linking spiral amplification, rapid detection, clinical specimens

## Abstract

PCRThere has been increasing interest in the head and neck squamous cell carcinoma (HNSCC) that is caused by high-risk human papillomavirus (HR-HPV) and has posed a significant challenge to Otolaryngologists. A rapid, sensitive, and reliable method is required for the detection of HR-HPV in clinical specimens to prevent and treat HPV-induced diseases. In this study, a multiple cross-linking spiral amplification (MCLSA) assay was developed for the visual detection of HPV-16. In the MCLSA assay, samples were incubated under optimized conditions at 62°C for 45 min, and after mixing with the SYBR Green I (SGI) dye, the positive amplicons showed bright green fluorescence while the negative amplicons exhibited no obvious change. The specificity test revealed that the developed MCLSA technique had high specificity and could effectively distinguish all five HPV-16 strains from other pathogenic microorganisms. In terms of analytical sensitivity, the limit of detection (LoD) of MCLSA assay was approximately 5.4 × 10^1^ copies/tube, which was 10-fold more sensitive than loop-mediated isothermal amplification (LAMP) and RT-PCR. The detection results of laryngeal cancer specimens collected from 46 patients with suspected HPV infection in the Liaoning region demonstrated that the positive detection rates of MCLSA and hybridized capture 2 kit were 32.61% (15/46). The true positive rate of the MCLSA assay was higher than that of RT-PCR (100% vs. 93.33%) and LAMP (100% vs. 86.67%). Therefore, the MCLSA assay developed in the present study could be a potentially useful tool for the point-of-care (PoC) diagnosis of HR-HPV, especially in resource-limited countries.

## Introduction

Over the past two decades, there has been a continuous increase in the incidence of head and neck squamous cell carcinoma (HNSCC) worldwide despite a decline in traditional risk factors, such as tobacco and alcohol use [1, 2]. The HNSCC represents a spectrum of tumors, including the ones in the oral cavity, pharynx, nasal cavity, and larynx, which account for 6% of all malignant tumors, with more than 600,000 newly diagnosed cases of HNSCC worldwide every year [3, 4]. The epidemiologic studies have implicated that persistent infection with high-risk Human papillomavirus (HR-HPV) could be a major risk factor and the likely etiologic agent for the subsequent development of HNSCC. Among the different HR-HPV types that can infect humans, HPV subtypes 16 and 18 are associated with approximately 70% of HNSCC cases [5, 6]. There is an urgent requirement for the application of an HR-HPV detection method for primary screening in clinics for the treatment as well as describe the prognosis of HNSCC patients with potential for treatment selection based on tumor HPV status [7, 8].

Currently, there is no “gold standard” method for the diagnosis of HPV infection in a strict sense. The methods used still rely on molecular biology techniques from the late 1980s that use nucleic acid probes. These techniques have cumbersome operation; they use nucleic acid probes labeled with ^32^P radioactive phosphorus; they are unable to confirm all carcinogenic HPV genotypes; thus, they have not been widely used in the early stages [9, 10]. Commercial HPV detection kits (such as Hybrid Capture 2 kit developed by Qiagen Corporation) can detect almost all types of carcinogenic HR-HPVs, as well as most low-risk noncarcinogenic HPV genotypes [11]. However, due to patent protection, it is an expensive method. Besides, various PCR-based detection methods are also widely used for HPV detection, genotyping, and viral load determination. These methods include blot hybridization, Papillo Check, and quantitative real-time PCR (qRT-PCR). These methods have the ability to rapidly achieve the specified amplification to HPV and virus genotyping and can generate viral load (concentration) data from the reaction curve generated by RT-PCR reaction kinetics [10, 12]. However, the PCR-based techniques are complicated, time consuming, and involve a thermal cycling for heating and cooling [13]. Thus, there is still a need for a rapid, cost-effective, and precise method to determine HR-HPV infection status in HNSCC.

Over the years, several isothermal amplification approaches (IAAs) have been developed for the amplification of DNA or RNA, such as loop-mediated isothermal amplification (LAMP) [14], helicase-dependent amplification (HDA) [15], Nucleic acid sequence-based amplification (NASBA) [16], cross-priming amplification (CPA) [17], Rolling circle replication (RCR) [18], multiple cross displacement amplification (MCDA) [19], and isothermal multiple self-matching-initiated amplification (IMSA) [20], etc. These detection techniques are simple, inexpensive, and rapid, and thus, fulfill the key criteria for the “on-site” diagnosis, as well as obviate the use of thermal cycling apparatus that has been widely used in point-of-care (PoC) testing. However, these IAAs suffer from several limitations, such as the need for rigorous optimization, the use of two or more enzymes (*e.g.*, HDA and NASBA require multiple enzymes), and/or special reagents. Additionally, they have drawbacks related to their operability, sensitivity, and specificity. Although LAMP, RCA, and CPA assays are rapid and sensitive, and can efficiently achieve amplification using only one enzyme (*Bst*, *Bsm*, or *GspSSD* polymerase), they might provide some percentage of false positive results due to the presence of marginal amounts of nucleic acid sequences [21]. In this study, we developed a novel IAA, named multiple cross-linking spiral amplification (MCLSA) assay to overcome the technical difficulties posed by current isothermal nucleic acid amplification techniques. This novel methodology combines ASSURED: affordable (a single enzyme is used), sensitive (amplification of trace DNA), specific (identifying multiple sequences of targets), user-friendly (result read simplification), rapid/robust (completing the reaction within 90 min), equipment-free (getting rid of the limitation of thermal cycler), and deliverable (test strip for mass production), and thus, meets the demand of rapid molecular analysis in clinical research. Here, we illustrate the optimization and application potential of the basic MCLSA assay. As a proof of concept, we detected HPV16 by the MCLSA method to demonstrate the capability of target analysis. The performance of the MCLSA assay in detecting HR-HPV from clinical samples was successfully evaluated; thus, providing a new opportunity for the development of HR-HPVs diagnostic methods.

## Materials and Methods

### Clinical Specimen Collection, Plasmids, and DNA Preparation

We collected specimens from 46 patients with biopsy confirming HNSCC without distant metastasis at the Shengjing Hospital of China Medical University between May 2019 and March 2020 for the development of this assay. All biopsy specimens were taken from two lesions (one was used for routine pathological diagnosis, and the other for DNA extraction), each 3 mm in diameter. We collected a written informed consent form from all participants. Also, age and gender of the subjects were recorded. The classification of tumor, node, and metastasis (TNM) of HNSCC was done according to the AJCC Staging Manual (8^th^ Ed., 2017). The protocol was approved by the Institutional Review Board and The Human Ethics Research Committee of China Medical University (No: 2020PS223K). Additionally, a complete genome sequence of HPV-16 (approximately 7,906 bp) was obtained from GenBank, synthesized, and cloned into the pAR2 expression vector (named pHPV-16), which was maintained in the Top10 *E. coli* was used as the positive control to determine the specificity and sensitivity of MCLSA. Other published reference strains (including HPV-18, HPV-31, pHPV-35a, pHPV-45, norovirus, etc.; Table 1) were used as negative controls and purchased from BeNa Culture Collection (BNCC), China. DNAs were prepared by extracting the genomic DNA from the standard strains using PureLink Viral RNA/DNA mini kits (Thermo Fisher scientific, China). Then the DNAs concentrations were measured using a NanoDrop Spectrophotometer at 260 nm and stored at -80°C until further analysis.

### Design of Primers for MCLSA Assay

**Fig. 1:**
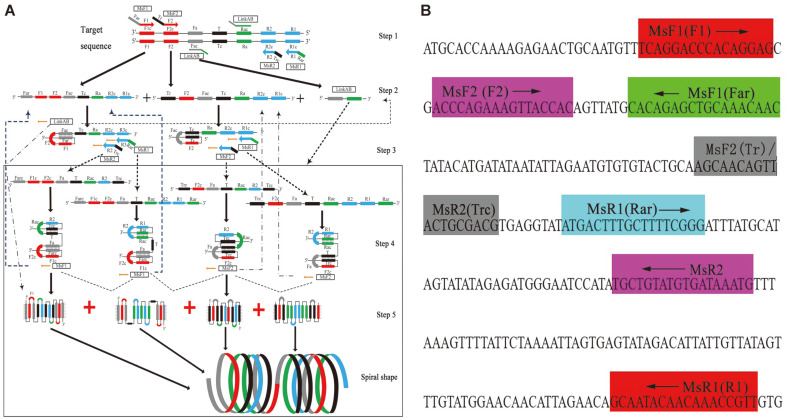
Schematic and primers location of MCLSA assay. **A**: Schematic diagram of MCLSA. **B**: Binding location for MCLSA specific primers. The arrows indicate the direction of primers extension.

We designed a set of MCLSA-specific primers targeting seven distinct regions based on the nucleotide sequence of the E6/E7 protein gene nucleotide sequence in HPV-16 (the NCBI GenBank database (No. NC_001526)) using Primer Premier 5.0 (PREMIER Biosoft International Co., USA). The specificity of the MCLSA primer set was confirmed via Blast analysis. Fig. 1 and Table 2 show the primer design, sequences, and locations of MCLSA primers. The specific primer design principle was similar to that applied in other IAAs but included two outer-spiral primers (MsF1/MsR1) with 5’ terminal Far and Rar sequences reverse to corresponding target sequence (Fa and Ra), while the 3’ sequence of outer-spiral primers (F1 and R1) corresponded to the F1c and R1c. Two inner-spiral primers (MsF2/MsR2) with 3’ terminal F2 and R2 were complementary to the F2c and R2c, while the Tr and Trc sequences at the 5’ terminal were complementary to the target sequences. LinkAB (comprising of Fa and Ra) was exclusively complementary to Fac and Rac sequence to further complete the displacement acceleration process of amplification. All the oligomers were synthesized and purified by GENEWIZ Biotech Ltd, China to provide high-performance liquid chromatography (HPLC) grade products. Furthermore, primers used for the LAMP assay and RT-PCR of HPV-16 were synthesized as previously described to assess the specificity and sensitivity of this MCLSA assay [7, 22].

### Reaction Conditions and System Optimization

The MCLSA reaction was mainly based on the conventional system used in other IAAs and performed in a 25 μl reaction mixture containing the following components: 2.0 μl each MsF1/MsR1 (10 mM) and MsF2/MsR2 (10 mM) primers, 1.0 μl LinkAB (10 mM) primers, 2.5 μl 10× Bst ThermolPol-buffer (New England Biolabs, USA), 2.5 μl (10 mM) of dNTPs (Takara Bio, China), 2.0 μl (3 mM) of MgCl_2_ (Aobox, China), 2.0 μl (1.0 M) of betaine (Sigma-Aldrich, Germany), 1 μl of 8 U Bst DNA polymerase large fragment (New England Biolabs), 2.0 μl of an appropriate concentration of standard DNA (approximately 30 ng), and 4.0 μl of sterilized deionized water. The reactions were conducted at 65°C for 60 min, followed by incubation at 85°C for 5 min to terminate the reaction. Apart from visual reading, a special closed isothermal amplification tube (cIAT, Guangzhou Hua-feng Biological Co., Ltd, China) was combined with the visual SYBR Green I (SGI, China) dye to complete the color development process visualized by eye or under UV light. The MCLSA products were further assessed using ethidium bromide (0.5 μg/ml) in stained agarose gel (2.0%), to confirm the accuracy of the observed fluorescence. Additionally, we optimized the reaction conditions by gradient under different reaction conditions (incubation temperatures and time) and system components (Mg^2+^, dNTPs, betaine, and Bst DNA polymerase) to establish the effectiveness of this highly efficient MLCSA approach. Table 3 shows the specific optimization scheme.

### The Analytical Specificity of the MCLSA Assay

We tested five strains of HBV (pHPV-16 and four clinical isolates) and 12 non-HPV-16 standard strains (pHPV-18, pHPV-31, pHPV-35a, pHPV-45, norovirus, *Mycobacterium tuberculosis*, *Staphylococcus* aureus, etc.) with MCLSA, LAMP, and RT-PCR assay under the conditions described above to assess the specificity of the MCLSA methodology. The deionized water was set as blank control and 2% agarose gel electrophoresis (AGE) was used to analyze the results or observe the color change in the cIAT tube directly under ultraviolet light to examine the amplification results of MCLSA and LAMP. Each sample was analyzed three times to ensure that there was no primer cross reactivity leading to false positive amplification.

### Evaluation of the sensitivity of the MCLSA assay

The pHPV-16 plasmid templates were 10-fold serially diluted with sterilized deionized water from an initial concentration of 5.4 × 10^7^ copies/tube to 5.4 × 10^-1^ copies/tube to conduct MCLSA, LAMP, and RT-PCR assay to determine the limit of detection (LoD) of the MCLSA assay. All reactions were performed as per the standardized protocols and same template DNA concentration. The analytical sensitivity of MCLSA and LAMP was determined using 2% AGE and SGI fluorescence dyestuff as previously described, while that using RT-PCR assay was confirmed based on the real-time fluorescence curve. All the three reaction assays were performed in triplicates to verify the analytical sensitivity.

### Examination of MCLSA Assay Using Clinical Specimens

We obtained 46 clinical laryngeal cancer specimens suspected of HPV infection from Liaoning province in China between 2019 and 2020 to evaluate the diagnostic accuracy of the HPV-16 MCLSA assay as a PoC surveillance tool. All the HPV-suspected specimens were extracted using commercial kits as previously described and then tested by a Hybrid Capture 2 High-Risk DNA kit following the manufacturer’s protocol (Qiagen Corporation, Inc.) as a reference standard. Then, we used 2 µl of each supernatant for MCLSA, LAMP, and RT-PCR assays, respectively. The detection rate of these three assays were calculated and compared using Hybrid Capture 2 kit to evaluate the applicability of the newly established MCLSA method.

## Results

### Development and Mechanism of the MCLSA Assay

The whole detection stage of MCLSA assay was mainly composed of two processes: the first stage relied on the isothermal amplification of the targeted DNA employing Bst DNA polymerase with strand displacement (Fig. 1A). In the MCLSA system, two pairs of spiral primers (MsF1/MsR1 and MsF2/MsR2) containing 5’ terminal reverse sequences corresponding to the target sequence simultaneously initiated the system amplification, when the incubation temperature reached 60-65oC. We used random amplification and a single strand in the target sequence as an example. Subsequently, the specific primers of MsR1 and MsR2 recognized the F1c and F2c fragments on the target sequence in the presence of Bst DNA polymerase, and then, formed DNA double strand structure with the extension to the 3’ end, while the whole process was in dynamic equilibrium with the single/double strand due to the presence of betaine (step 1-2). The R2/R3 segment of other two primers MsR2/MsR1 was combined with the R2/R3 segment of a single strand and extended to 5’ (Step 3). In pace with the reaction, the Farc (Trc) segment rotated and combined with the Fa (T) segment to form a series of complex spiral structures and continue to re-extend towards the 3’ end to form a longer spiral structure (step 4-5). Additionally, the LinkAB (cross-linking primer) ligated with the Fac/Rac to accelerate amplification and the synthesized products with complementary amplification could be renewed as new target sequences to continue the reaction.

**Fig. 2:**
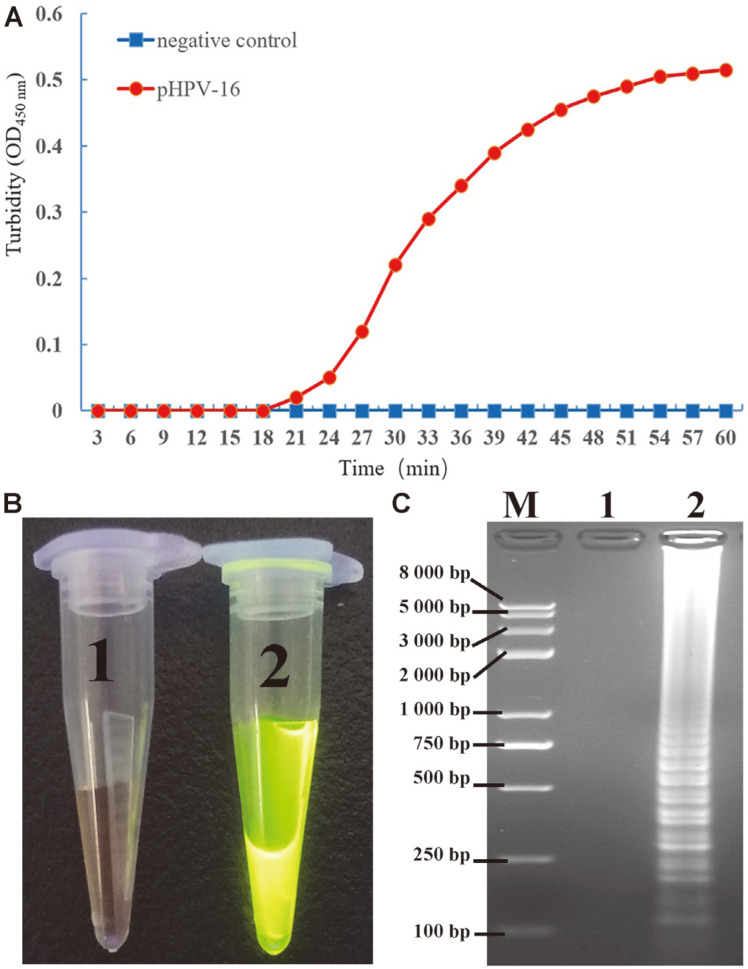
Detection results for the MCLSA-specific primers. **A**: Observation of real-time turbidity for the primer combinations of HPV-MCLSA. **B**: Observation of the MCLSA amplified products mixed with SGI dye under ultraviolet light. **C**: 2% AGE of the MCLSA amplified products. M: DNA maker DL8000. 1: negative control; 2: Amplification of pHPV-16.

The second stage involved the reading, detection, and visualization of results based on MCLSA amplicons: the MCLSA amplicons could be quickly visualized based on the turbidity changes of the mixture due to the high levels of pyrophosphate ion. As depicted in Fig. 2A, the samples of genomic DNA extracted from pHPV-16 strain was amplified by MCLSA assay from 20 min and the turbidity value increased gradually over time, while the negative control (sterilized deionized water) was negative for amplification. At the same time, the color change could be read directly with SGI chromogenic dye under ultraviolet light. The positive sample bright green fluorescence while the negative sample showed no change (Fig. 2B). Furthermore, these results were also confirmed by 2% AGE to form a characteristic DNA ladder band in positive samples stained with ethidium bromide (5 μg/mL), while the negative controls did not show this change (Fig. 2C).

### Optimization of the MCLSA Reaction System

**Fig. 3:**
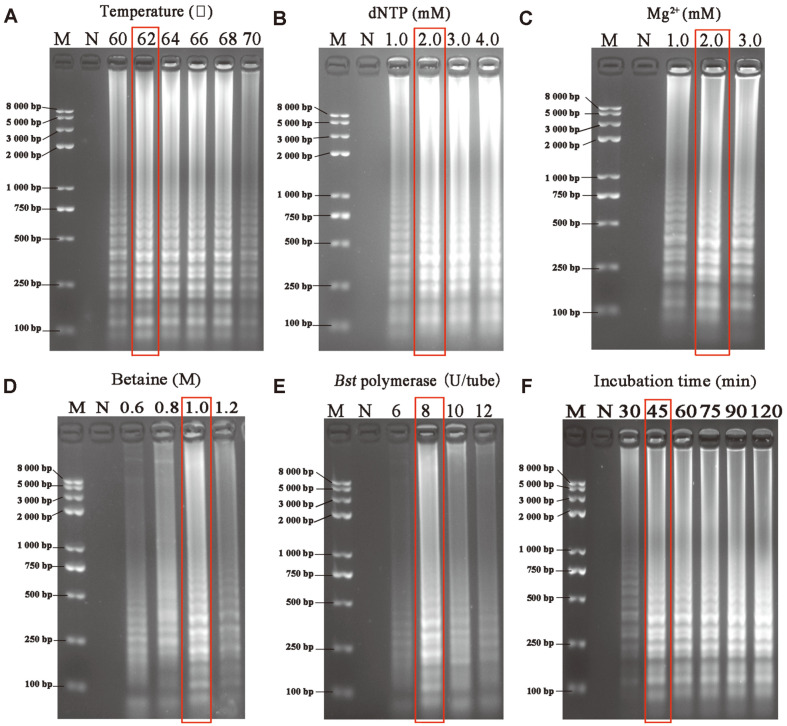
Optimization of MCLSA amplification conditions for the detection of pHPV-16 monitored by 2% AGE. Optimization of **A**: Incubation temperature. **B**-**E**: Concentration of dNTPs, MgCl_2_, betaine, and Bst DNA polymerase, respectively. **F**: incubation time. M: DNA maker DL8000. N: negative control.

The incubation temperature, incubation time, and the components (concentration of Bst DNA polymerase, Mg^2+^, dNTPs, and betaine solution) in the system were seriatim optimized using the 2% AGE to improve the amplification efficiency of the MCLSA assay. The pHPV-16 DNA was used as the template at the concentration of 5.4 × 10^7^ copies/tube to examine the optimal temperature; the electrophoretograms showed no obvious difference in the gradient bands produced at temperatures ranging from 62-68°C; However, based on the selection principle of high efficiency and low cost, 62oC was selected as the best temperature for detection (Fig. 3A). Based on the fixed incubation temperature of 62°C, we subsequently optimized the concentration of dNTPs, MgCl_2_, betaine solution, and Bst-DNA polymerase for 60 min and the final optimized conditions were as follow: 2.0 mM dNTPs, 2.0 mM MgCl_2_, 1.0 M betaine, and 8 U/tube of Bst DNA polymerase (Figs. 3B-E). Additionally, with the increase in reaction time, the electrophoresis band became more obvious and the best amplification effect was obtained at 45 min (Fig. 3F).

### Analytical Specificity of MCLSA Assay

**Fig. 4:**
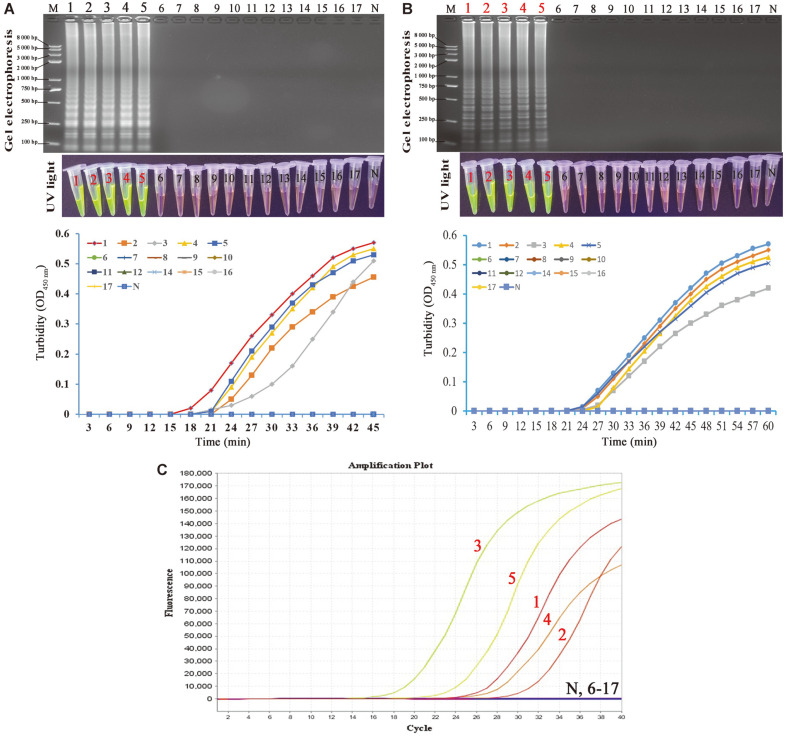
The specificity of MCLSA assay compared with LAMP and RT-PCR assay. **A**: The MCLSA amplicons were tested using different genomic DNA templates and observed by AGE, SGI under UV light and real-time turbidity monitoring, respectively. **B**: Observation of the production from LAMP assay using AGE, SGI under UV light and real-time turbidity monitoring, respectively. C: Amplification results of RT-PCR assay. M: DNA maker DL8000. N: negative control. 1- 5: pHPV-16, HPV-16-105, HPV-16-224, HPV-16-276, and HPV-16-335. 6-9: pHPV-18, pHPV-31, pHPV-35a, and pHPV-45; 10: Hepatitis B virus; 11: Hepatitis C virus; 12: norovirus; 13: norovirus; 14: *Mycobacterium tuberculosis*; 15: *Staphylococcus aureus*; 16: *Klebsiella pneumonia*; 17: *Salmonella*.

The specificity of the MCLSA assay was evaluated by testing the genomic DNA extracted from five HPV strains and 12 non-HPV strains, respectively (Table 1). We observed the visible fluorescence under ultraviolet light and ladder-like pattern in the agarose gel of MCLSA amplicons in all five of HPV-16 strains, while no significant change was observed in the negative control and non-HPV-16 strains (Fig. 4). The detection results demonstrated that the MCLSA assay had a 100% specificity for the identification of HPV-16, which were consistent with the results of the LAMP and RT-PCR assays.

### Analytical Sensitivity of MCLSA-LFB Assay

**Fig. 5:**
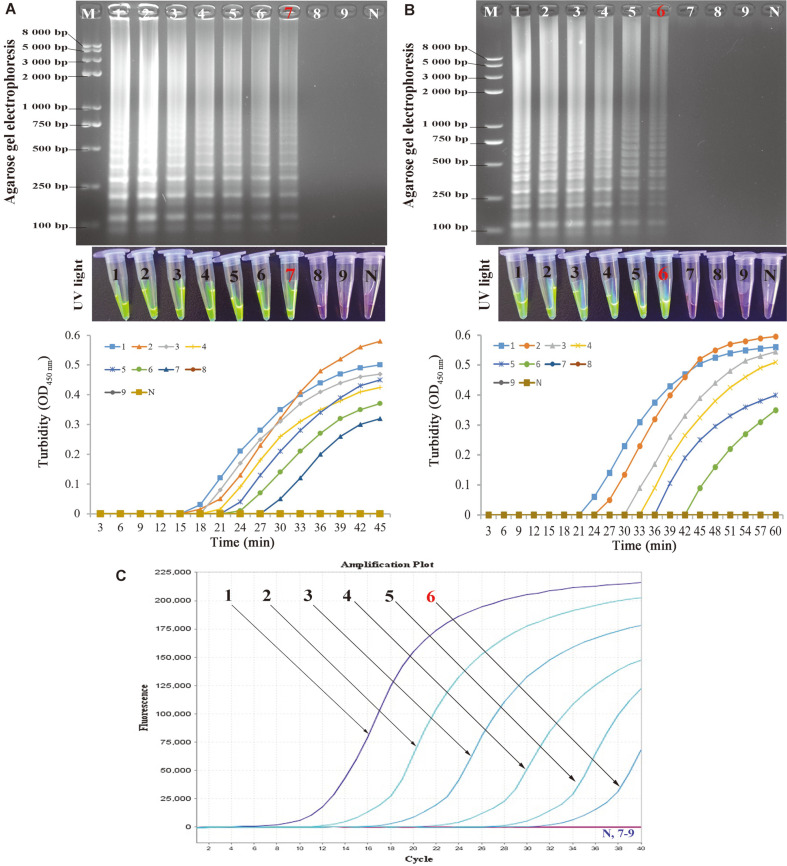
Sensitivity of the MCLSA assay for HPV-16 detection. **A**: Analysis of the MCLSA amplicons by AGE, SGI under UV light and real-time turbidity monitoring. **B**: Amplification results of LAMP assay by AGE, SGI under UV light and real-time turbidity monitoring. **C**: Detection of HBV by RT-PCR. Lanes/tubes/ fluorescence signals 1-9: 5.4 ×10^7^, 5.4 ×10^6^, 5.4 ×10^5^, 5.4 ×10^4^, 5.4 ×10^3^, 5.4 ×10^2^, 5.4 ×10^1^, 5.4 ×10^0^, 5.4 ×10^-1^ copies/tube, respectively. M: DNA maker DL8000. N: negative control.

We performed a 10-fold serial dilution (from 5.4 × 10^7^ copies/tube to 5.4 × 10^-1^ copies/tube) using sterilized deionized water for the known concentration of pHPV-16 genomic DNA by analyzing the amplicons in triplicates to further assess the LoD of the MCLSA assay compared with other molecular techniques (LAMP and real-time PCR assay) for the detection of HPV-16 (Fig. 5). The LoD of MCLSA was 5.4 × 10^1^ copies/tube of HPV-16 based on both 2% AGE or visual inspection with SGI dye, which was 10 times more sensitive than that of LAMP (5.4 × 10^2^ copies/tube) and RT-PCR (5.4 × 10^2^ copies/tube), respectively. These data indicated that the developed MCLSA assay was more sensitive and suitable for HPV-16 detection at the molecular level.

### Validation with Clinical Samples of MCLSA Assay

**Fig. 6:**
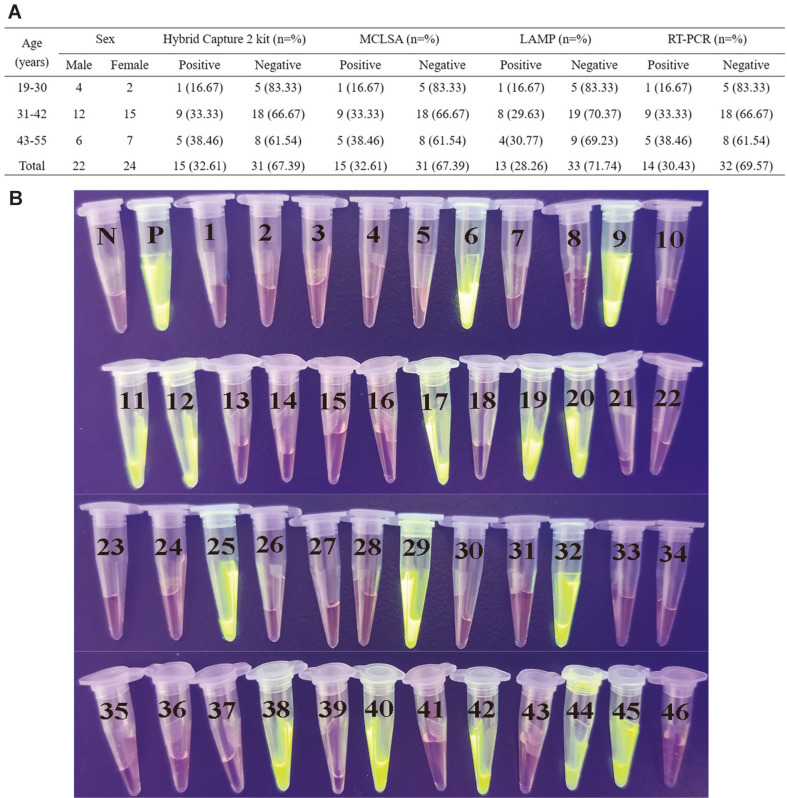
Visual MCLSA assay for the detection of HPV-16 in clinical samples. **A**: Detection results of 46 clinical specimens of suspected HPV-16 amplified by HC2, MCLSA, LAMP and RT-PCR. **B**: The results were visualized by mixing of SGI dye (1 μl) into the amplicons (25 μl) after the MCLSA reaction. 1-46: the detection results of MCLSA assay in 46 clinical samples with suspected HPV-16 infection. M: DNA maker DL8000. P: positive control (pHPV-16). N: negative control.

The extracted DNA from 46 HPV-suspected clinical laryngeal cancer specimens were analyzed by MCLSA, LAMP, and RT-PCR assay to determine the practicability of MCLSA method as a reliable surveillance tool for the HPV-16 in PoC (Fig. 6). According to the reference Hybrid Capture 2 kit method, 15 HPV-16-positive specimens were detected in the 46 clinical specimens, and the diagnostic accuracy was consistent with the MCLSA assay (32.61%). The detection results of LAMP and RT-PCR method identified that 13 (86.67%) and 14 (93.33%) positive specimens among 15 positive specimens, respectively (Fig. 6). These findings implied that the MCLSA assay was well suited as a credible tool in PoC field compared with the LAMP and RT-PCR assay.

## Discussion

Currently, HNSCC is the 6^th^ most common cancer worldwide with a high prevalence rate and mortality; thus, it is a public health issue, especially in underprivileged communities [23, 24]. Although the clinical data of HPV infection rate in LC patients are largely inconsistent, several molecular epidemiological studies have shown that HPV is strongly associated with laryngeal cancer morbidity [22, 25]. However, the current “gold standard” methods of diagnosis, such as Digene’s Hybrid Capture 2 kit or PCR, are expensive and time consuming, and require expensive equipments, making the screening and diagnostic processes very challenging, especially in primary medical units [10, 26]. With the enhanced understanding of the application of molecular biology, the development of numerous IAAs methods has promoted clinical screening and on-site diagnosis to overcome the difficulties associated with time-consuming and complex operation. Among the advanced IAAs methods, rapid detection of HPVs based on the LAMP approach is one of the frequently used methods, which uses two or three pairs of primers combined with DNA polymerase with chain replacement function to synthesize DNA amplicons of various lengths at constant temperatures. This isothermal amplification method has been found to be rapid, highly specific, and sensitive; thus, facilitating the popularization and industrialization of detection reagents, as well as the development of new IAAs. However, the LAMP technology has several limitations. First, LAMP amplification product detection is usually similar to the PCR technique, which requires AGE for observation. Since this reaction generates significant amount of aerosols, this open-tube detection method is known to cause experimental pollution. Second, although loop primers used by LAMP method increased its amplification efficiency, the nonspecific pairing between primers increased the probability of false-positive results during amplification. Third, the LAMP technique is a Japanese patented technology with intellectual property rights, and thus, is often subjected to patent restrictions during application and development.

Based on these observations, the MCLSA method developed here, combined the advantages of other IAAs with the relative simplicity of isothermal amplification with a design principle similar to the PCR assay. However, it used the Bst polymerase to amplify target sequence under isothermal condition to improve the efficiency of DNA amplification. All reagents and incubation conditions in the reaction system were initially optimized to establish the amplification system to achieve the optimal amplification state of the MCLSA reaction. The optimal amplification results were obtained at 62oC for 45 min with the following concentrations of dNTPs (2.0 mM), MgCl_2_ (2.0 mM), betaine (1.0 M), and Bst DNA polymerase (8 U/tube). Additionally, all isothermal amplification techniques were associated with the possibility of aerosol contamination. Once the reaction tube was opened for AGE analysis or dye-dropping visualization, there was a risk of aerosol contamination, especially in the field diagnostic environment. Therefore, we combined a special closed isothermal amplification tube (cIAT) with SGI dye which combined with the DNA double strand to eliminate false-positive results caused by aerosol leakage. Before the reaction, the mixture of SGI dye and amplification reagent were placed on both sides of the amplification tube, and after the reaction, the SGI on one side was slowly mixed with the amplification product on the other side. The positive amplicons showed bright green fluorescence, while the negative amplicons remained orange. Simultaneously, DNA extraction, MCLSA reaction, and electrophoresis were performed in isolation to reduce sample contamination and false-positive results. In terms of detection cost, we calculated the operation size of the MCLSA assay and RT-PCR assay, based on 20 samples in each analysis format, including reagents, depreciation of equipment, and $2/hour salary for technicians. The MCLSA has a considerable economic advantage for the identification of HPV-16 compared with the RT-PCR assay ($3.8 vs $7.3 per reaction). Also, the entire MCLSA procedure, including DNA extraction (20-25 min), sample loading and isothermal amplification (60 min), and result reading (5 min), was accomplished within 90 min, without requiring complicated instruments and labor costs.

In this experiment, we designed the primers of MCLSA assay targeting the E6/E7 promotor region of the HPV-16 genome, which has the conserved nucleotide sequence among individual HPV subtypes and has been successfully used as a target in LAMP protocols [7]. Meanwhile, the specificity of MCLSA assay was successfully confirmed with genomic DNA of all the HPV-16 positive isolates without any cross-reaction whereas all non-HPV-16 strains displayed negative results. These results were consistent with those of the LAMP and RT-PCR assay, either based on the AGE analysis or SGI-based dye method. In the case of sensitivity, the MCLSA proposed here could detect as little as 5.4 × 10^1^ copies per reaction of HPV-16 DNA, which was 10-fold more sensitive than the LAMP and qPCR assay. Interestingly, we obtained a lower LoD values when amplified using the previously reported LAMP primers [7], which sensitivity was similar to that of the LAMP combined with lateral flow dipstick tests reported by Kumvongpin et al [13]. Factors such as professional operators, changes in chemical reagents, and primer purity will affect the slight changes of amplification results to some extent. However, the sensitivity of the MCLSA method established in this study was significantly higher (approximately 1 to 3 orders of magnitude) than that reported in recent years for the diagnosis of HPV-16 using LAMP method [26, 27]. The data presented here demonstrated that the MCLSA assay is a rapid, highly sensitive, and robust diagnostic method for the identification of HPV-16.

The clinical applicability of MCLSA assay was tested on 46 HPV-16 suspected clinical samples compared with LAMP, RT-PCR, and Hybrid Capture 2 kit assays. The test results indicated that the MCLSA and Hybrid Capture 2 kit methods could adequately detect the HPV-16 in the LC samples, and displayed higher detection sensitivity for the target virus compared with the LAMP and RT-PCR assay (Table 3). All these results demonstrated that MCLSA assay had high screening ability for HPV-16 DNA, which could act as a potential tool for HPV identification with high specificity and sensitivity. Until now, although many isothermal techniques have shown great promise for the detection of pathogenic microorganisms and, also provided several alternatives for large-scale clinical screening. However, these IAAs have some limitations, such as they need specific primers designed for a limited length of gene fragment (300bp) to ensure the accuracy of detection. Meanwhile, with the increase of the sensitivity of this technique, there will be a substantial increase in the false-positive cases of aerosol-related contamination. Further research is required to develop methods to completely avoid this issue. As a new member of the IAAs family, the MCLSA method established here exhibited higher sensitivity compared with the LAMP and RT-PCR method, and provides a simple, less time-consuming and highly sensitive supplementary method for HPV monitoring in resource-limited areas.

## Figures and Tables

**Table 1:** Strains used in this study.

Strain	Source^1^	Method^2^		

		MCLSA	LAMP	RT-PCR
pHPV-16	Synthesized	+	+	+
HPV-16-105	Self-isolate	+	+	+
HPV-16-224	Self-isolate	+	+	+
HPV-16-276	Self-isolate	+	+	+
HPV-16-335	Self-isolate	+	+	+
pHPV-18	ATCC 45152D	－	－	－
pHPV-31	ATCC 65446	－	－	－
pHPV-35a	ATCC 40330	－	－	－
pHPV-35b	ATCC 40331	－	－	－
Hepatitis B virus	ATCC HB-8065	－	－	－
Hepatitis C virus	ATCC 40602	－	－	－
Norovirus	BNCC 300545	－	－	－
Norovirus	BNCC 254071	－	－	－
*Mycobacterium tuberculosis*	ATCC 25177	－	－	－
*Staphylococcus* aureus	ATCC 25923	－	－	－
*Klebsiella pneumoniae*	ATCC 70603	－	－	－
*Salmonella*	ATCC 13076	－	－	－

^1^ATCC = American Type Culture Collection; BNCC= Bena Culture Collection; Self-isolates were preserved in our lab.

^2^MCLSA = multiple cross-linking spiral amplification; LAMP = loop-mediated isothermal amplification; RT-PCR = real-time PCR; + = positive result; − = negative result.

**Table 2:** Primers of MCLSA, LAMP and RT-PCR assays.

Name	Primer ID	Primers Sequence (5'-3')	Nucleotide Length
MCLSA	MsF1	GTTGTTTGCAGCTCTGTG-TCAGGACCCACAGGAG	34
	MsR1	GGGCTTTTCGTTTCAGTA-AACGGTTTGTTGTATTGC	36
	MsF2	CGTCGCAGTAACTGTTGCT-ACCCAGAAAGTTACCA	35
	MsR2	GCAGCGTCATTGACAACGA-CATTTATCACATACAGCA	37
	LinkAB	CACAGAGCTGCAAACAACgggcccATGACTTTGCTTTTCGGG	42
LAMP^[7]^	FIP	TGGGGCACACAATTCCTAGT-CACACACGTAGACATTCGT	39
	BIP	TCAGAAACCATAATCTACCATGGC-ATTACATCCCGTACCCTCTT	44
	F3	TCGGTTGTGCGTACAAAG	18
	B3	AGCCTCTACATAAAACCATCC	21
	LF	CCCATTAACAGGTCTTCCAAAGT	23
	LB	CCTGCAGGTACCAATGGGG	19
RT-PCR^[22]^	E6-F	GAGAACTGCAATGTTTCAGGACC	23
	E6-R	TGTATAGTTGTTTGCAGCTCTGTGC	25
	TaqMan probe	CAGGAGCGACCCAGAAAGTTACCACAGTT	29

**Table 3:** Optimization of MCLSA assay for detection of HPV-16.

Conditions	The test interval	Difference	Good yield	Final selected^a^
Temperature	60 - 70 ℃	2 ℃	62 - 68 ℃	62 ℃
MgCl_2_	1.0 - 3.0 mM	1.0 mM	2.0 - 3.0 mM	2.0 mM
dNTPs	1.0 - 4.0 mM	1.0 mM	2.0 mM	2.0 mM
Betaine	0.6 - 1.2 M	0.2 M	1.0 M	1.0 M
*Bst* polymerase	6 - 12 U	2 U	8 U	8 U
Incubation time	30 - 120 min	15 min	45 - 120 min	45 min

^a^The final concentrations of reagents or temperature and times used in MCLSA assay.
